# Functional traits predict resident plant response to *Reynoutria japonica* invasion in riparian and fallow communities in southern Poland

**DOI:** 10.1093/aobpla/plab035

**Published:** 2021-06-03

**Authors:** Marcin W Woch, Paweł Kapusta, Małgorzata Stanek, Szymon Zubek, Anna M Stefanowicz

**Affiliations:** 1 Institute of Biology, Nicolaus Copernicus University in Toruń, Lwowska 1, PL- 87-100 Toruń, Poland; 2 Władysław Szafer Institute of Botany, Polish Academy of Sciences, Lubicz 46, PL-31-512 Kraków, Poland; 3 Institute of Botany, Faculty of Biology, Jagiellonian University, Gronostajowa 3, PL-30-387 Kraków, Poland

**Keywords:** Invasive plant species, *Reynoutria japonica*, community structure, plant traits, interspecific competition, soil properties, seasonal variability

## Abstract

*Reynoutria japonica* is one of the most harmful invasive species in the world, dramatically reducing the diversity of resident vegetation. To mitigate the impact of *R. japonica* on ecosystems and properly manage affected areas, understanding the mechanisms behind this plant’s invasive success is imperative. This study aimed to comprehensively analyse plant communities invaded by *R. japonica*, taking into account species traits, habitat conditions and seasonal variability, and to determine the ecological profile of species that withstand the invader’s pressure. The study was performed in fallow and riparian areas in southern Poland. Pairs of adjacent plots were established at 25 sites with no obvious signs of recent human disturbance. One plot contained *R. japonica*, and the other contained only resident vegetation. For each plot, botanical data were collected and soil physicochemical properties were determined. Twelve sites were surveyed four times, in two springs and two summers, to capture seasonal variability. The presence of *R. japonica* was strongly associated with reduced resident plant species diversity and/or abundance. In addition to the ability to quickly grow and form a dense canopy that shades the ground, the success of the invader likely resulted from the production of large amounts of hard-to-decompose litter. The indirect impact of *R. japonica* by controlling the availability of nutrients in the soil might also play a role. A few species coexisted with *R. japonica*. They can be classified into three groups: (i) spring ephemerals – geophytic forbs with a mixed life history strategy, (ii) lianas with a competitive strategy and (iii) hemicryptophytic forbs with a competitive strategy. Species from the first two groups likely avoided competition for light by temporal or spatial niche separation (they grew earlier than or above the invasive plant), whereas the high competitive abilities of species from the third group likely enabled them to survive in *R. japonica* patches.

## Teaser

*Reynoutria japonica*, native to East Asia, is a highly invasive plant beyond its range. It easily outcompetes and displaces most species from plant communities it invades. However, there are some plant species that, due to their functional traits, can coexist with *R. japonica* and even benefit from its invasion. In our study, we found such species among spring ephemerals (they undergo a full vegetation cycle before the development of *R. japonica* canopy), lianas (they treat shoots of *R. japonica* as support for climbing and overgrow them) and highly competitive native plants.

## Introduction

Japanese knotweed (*Reynoutria japonica*) is one of the world’s most problematic invasive species (Lowe *et al*. 2000; EPPO 2020). This species, which is native to Asia, was introduced into Europe in the 19th century. This is an ecologically plastic, clonal, fast-growing, herbaceous, perennial geophyte that dramatically reduces the occurrence of native species, thus causing biotic homogenization (Maurel *et al*. 2010; Olden *et al*. 2016; Lavoie 2017). *Reynoutria japonica* inhabits mostly river banks and various types of wastelands. It is particularly problematic in riparian zones, where it poses a threat to resident communities and habitats with special nature conservation status (the Natura 2000 ecological corridors), and to the economy (Tokarska-Guzik *et al*. 2006; Gerber *et al*. 2008; Claeson and Bisson 2013). Once introduced, *R. japonica* spreads rapidly and forms dense stands often extending over several hundred square metres, eliminating many plant species or reducing their abundances to a few individuals (Gerber *et al*. 2008; Pyšek *et al*. 2009; Aguilera *et al*. 2010; Lavoie 2017). Its monodominant stands are sparse to widespread, and can be found from lowlands to the submontane areas of temperate climate zones in Europe (EPPO 2020).

Despite numerous studies, the reasons for the great competitive success of *R. japonica* are still unclear. It is usually attributed to a high growth rate and shading (Tokarska-Guzik *et al*. 2006; Aguilera *et al*. 2010; Maurel *et al*. 2010; Moravcová *et al*. 2011), production of large amounts of biomass characterized by low nutritional quality and the slow decomposition of this biomass (Aguilera *et al*. 2010; Tanner and Gange 2013; Mincheva *et al*. 2014; Stefanowicz *et al*. 2020), high regenerative capacity (de Waal 2001; Bímová *et al*. 2003; Dauer and Jongejans 2013), the ability to grow at low nutrient levels and in variable environments (Adachi *et al*. 1996; Parepa *et al*. 2013a), and allopathic effects (Moravcová *et al*. 2011; Murrell *et al*. 2011; Tharayil *et al*. 2013; Dommanget *et al*. 2014; Parepa and Bossdorf 2016). The success of this plant can also be determined by external factors such as environmental disturbances, including disturbances in the soil microbiome or the nutrient cycle (Dassonville and Guillaumaud 2011; Suseela *et al*. 2016; Zubek *et al*. 2016; Abgrall *et al*. 2018; Čerevková *et al*. 2019).

Functional diversity, i.e. the diversity of traits represented by species present in the ecosystem, is a key driver of ecosystem processes, and the basis of ecosystem resilience to environmental changes (e.g. disturbances) and reliability in delivering ecosystem services (Folke *et al*. 2004; Hooper *et al*. 2005; Laliberté and Legendre 2010). The functions and life-histories of species (traits) determine not only whether they are able to establish and survive in a given place, but also whether they can coexist with other species and become part of a larger whole, i.e. a community (Kempel *et al*. 2013; Kraft and Ackerly 2014; Kraft *et al*. 2015). The analysis of traits is crucial in understanding the mechanisms of coexistence of community members, but also the mechanisms of resistance of the resident community to invasion by alien species, which is crucial for mitigating the effects of this invasion and for effective ecosystem restoration following invader removal. According to some authors (Kraft *et al*. 2015; Fried *et al*. 2019), resident species are more likely to survive and coexist with the invader if they have similarly high abilities to compete for resources (competitive hierarchy) or if they have different resource use strategies (high niche differences). As for *R. japonica*, its invasion does not affect the resident plants equally. Some of them are quickly outcompeted by the invader, while others (less numerous) withstand its pressure and persist for a long time (although often in small abundances) in its dense stands (Tokarska-Guzik *et al*. 2006; Woch *et al*. 2019). These observations lead to the question to what extent the presence of resident plant species in *R. japonica* patches is determined by chance, and to what extent by their traits or trait combinations. Our study addressed this question.

Identifying and quantifying the importance of between-species relationships determining the success of alien or resident species is difficult because environmental factors interact with species characteristics (Kempel *et al*. 2013). Therefore, it is crucial to control relevant environmental variables in invasion research. Our study is the first to link the diversity and composition of resident communities, both affected and unaffected by *R. japonica*, with soil physicochemical properties, taking into account seasonal variability.

The aims of our study were to: (i) characterize differences in the resident plant community structure (species richness and composition) resulting from the invasion by comparing *R. japonica* patches and adjacent (unaffected) vegetation patches, (ii) link the occurrence of resident plants with soil properties and the season (early spring vs. late summer) and (iii) identify functional traits that enable resident plants to coexist with *R. japonica*.

## Materials and Methods

### Reynoutria japonica

*Reynoutria japonica* is a perennial geophyte, which reproduces mainly vegetatively through the growth and regeneration of rhizomes and shoots. In early spring (late March or early April, depending on weather conditions), the rapid growth of annual shoots begins and continues until early summer. The growth rate is very high, from 3 cm/day in the initial growth period to 5–8 cm/day in the second half of May (Tokarska-Guzik *et al*. 2015). Shoots reach a height from 100 to 300 cm, averaging 150–200 cm, and form numerous branches on which leaves develop. Fully developed compact canopy occurs from June to September **[see****Supporting Information—Fig. S1A**–**C****]**. In October, the leaves begin turning yellow and fall, and then the rest of the above-ground biomass dies. The buds hibernate at the base of the shoot clumps and develop into the new shoots next spring **[see****Supporting Information—Fig. S1D****]**.

### Study sites and sampling

This study was conducted in western Małopolska and eastern Silesia (southern Poland) between the towns of Dąbrowa Górnicza, Wadowice, Katowice and Kraków. This area lies in the transitional climate zone between a temperate oceanic climate in the west and a temperate continental climate in the east. The average annual air temperature fluctuates between 7.1 and 8.1 °C, and the average annual rainfall fluctuates between 700 and 873 mm. Precipitation is the highest in June, July and August, and the lowest in February and March. Southwesterly winds are most frequent, followed by northeasterly winds. The growing season lasts 205 to 215 days (Lorenc 2005).

Twenty-five study sites were established in riparian zones of the Skawa, Soła and Vistula rivers, and in fallows, in places where *R. japonica* formed large and compact patches **[see****Supporting Information—Fig. S1A****]**. The sizes of these patches were estimated using aerial photographs. Each study site consisted of two paired plots, 4 m^2^ each – one located in a patch with *R. japonica* (90–100 % cover) and one in adjacent resident vegetation – giving a total of 50 plots. The plots in a pair (invaded plot and uninvaded plot) were placed a few metres from each other (approx. 4–6 m between the edges of the plots) in patches with a similar disturbance history to obtain the highest possible habitat similarity and at the same time to minimize the impact of *R. japonica* on the uninvaded plot, and vice versa, the impact of resident plant community on the invaded plot.

### Resident plant communities

Resident plant communities were identified according to Chytrý (2009, 2013) based on the plant species composition of the uninvaded plots. The total vegetation coverage in these communities was 100 %, with the exception of two plots, where 90 % coverage was recorded. The most common type of vegetation was the *Petasition hybrydi* Sillinger 1933 alliance (the *Galio-Urticetea* Passarge ex Kopecký 1969 class) – treeless vegetation, largely formed by flooding. It occurred in sites that were located in humid riparian zones, both natural and secondary habitats (e.g. wet meadows and arable fields abandoned ca. three decades ago); there were 20 such sites. The species with the highest frequency and coverage were *Phalaris arundinacea*, *Rubus caesius*, *Aegopodium podagraria*, *Petasites hybridus*, *Urtica dioica*, *Chaerophyllum aromaticum* and *Anthriscus sylvestris*. The community was also characterized by the presence of *Calystegia sepium*, *Allium ursinum*, *Ficaria verna*, *Humulus lupulus* and *Symphytum tuberosum*.

Another type of community was the *Convolvulo arvensis-Elytrigion repentis* Görs 1966 alliance (the *Artemisietea vulgaris* Lohmeyer *et al*. ex von Rochow 1951 class). It occurred in drier habitats (compared with riparian sites) – fallows (*N* = 5). Among the species with high frequency and coverage were *Calamagrostis epigejos*, *Agrostis stolonifera* and *Elymus repens*. Apart from that, *Cirsium arvense*, *Convolvulus arvensis* and *Artemisia vulgaris* were usually present in this community.

### Collection and handling of vegetation data

Within each plot, vascular plant species were recorded using the 12-point cover-abundance scale (1: <0.2 % cover and one small individual; 2: 0.2–1 % and one to three small individuals; 3: 1–3 % and two to five individuals; 4: 3–5 % and three to eight individuals; 5: 5–10 % and eight to 20 individuals; 6: 10–20 %; 7: 20–30 %; 8: 30–40 %, 9: 40–55 %; 10: 55–70 %; 11: 70–85 %; 12: 85–100 %). The plots were visited several times during the 2017–2018 growing seasons to obtain a complete list of species. The species nomenclature followed Mirek *et al*. (2002). In this study, all species except *R. japonica* were included in the group of resident plants. This was also the case with *Echinocystis lobata*, a plant invasive in Poland. This means that the term ‘resident plants’ cannot be regarded as strict, but for convenience, it is used throughout this paper.

Of the 25 study sites, 12 were selected to observe the between-season variability in the plant species occurrence. The plots (24 in total) within these sites were surveyed four times: summer 2017 (August 28 to September 1), spring 2018 (April 23 to 24), summer 2018 (August 22 to 24) and spring 2019 (April 23 to 24). In the case of one site, we managed to sample only twice, in summer 2017 and spring 2018, because the patch of *R. japonica* was disturbed by shoot-cutting.

The total number of vascular plant species (species richness), the total coverage and the number of species representing each plant trait were calculated. Before the calculations, the data from the four surveys (12 sites) were combined within the season; of the two cover-abundance values recorded for a given species, the higher was selected. The following traits were taken into account in this study: C-S-R life strategy (Grime 2001), life form (Raunkiaer 1934), phenology (i.e. whether the species was a spring ephemeral or not; Rutkowski 2008), as well as the functional group (forbs, graminoids, legumes, woody plants) and the plant community class to which the species belonged (Chytrý 2007, 2009, 2013). Additionally, for each plot, the values of two indices were determined: herb-layer disturbance frequency index (HDFI), calculated as the mean of the common logarithm of the disturbance frequency of all vegetation classes weighted by the occurrence frequencies of a given species in those classes, and herb-layer disturbance severity index (HDSI), defined as the mean disturbance severity of all vegetation classes weighted by the occurrence frequencies of a given species in those classes (Herben *et al*. 2016). Disturbance indices for particular plant species, which were necessary to calculate HDFI and HDSI, were taken from the supporting information to the work by Herben *et al*. (2016). *Reynoutria japonica* was excluded from all the above calculations, since it was hypothesized to be the cause of (not part of) the studied patterns.

### Soil properties

In August/September 2017, three subsamples (approx. 20 × 20 × 20 cm) of the organo-mineral soil horizon (A horizon) were taken from each plot and bulked to obtain one composite sample per plot. In the *R. japonica* plots, the thickness of the organic (O) horizon was determined; in the resident vegetation plots, the O horizon was almost absent, therefore it was not measured.

Soil samples were analysed for a number of physicochemical properties; the methods of analysis and the obtained data (descriptive statistics for invaded and uninvaded plots) were published elsewhere (Stefanowicz *et al*. 2020). For this study, 15 variables were selected: soil particle composition (sand, silt, clay), moisture, pH, the contents of organic C and total N, P, K and Ca, the concentrations of N-NH_4_, N-NO_3_, P-PO_4_ as well as the C/N and C/P ratios **[see****Supporting Information—Table S1****]**.

### Statistical analysis

Due to the fact that the datasets in this study consisted of many variables, often correlated with each other, the statistical analysis relied mainly on multivariate methods. Before multivariate analyses, the data were transformed to reduce variability and approximate normality: soil properties and vegetation parameters were log-transformed and then normalized, while species abundances were square-root transformed (Anderson *et al*. 2008).

The differences in soil physicochemical properties, resident plant species composition and vegetation parameters between the invaded plots and the uninvaded plots were determined using permutational multivariate analysis of variance (PERMANOVA). PERMANOVA models included in addition to the fixed effect (plot type) a random effect (site identifier). In the case of soil variables and vegetation parameters, PERMANOVA was performed based on Euclidean distances, while in the analysis of species data, Bray–Curtis distances were used. Sites with empty plots (no species other than *R. japonica*) were excluded from the analysis due to the impossibility to calculate Bray–Curtis distances (species data) and HDFI and HDSI indices (vegetation parameters) for them. For the species data, in addition to PERMANOVA, similarity percentage (SIMPER) analysis (Clarke 1993) using Bray–Curtis distances and with a 70 % contribution cut-off point was carried out to identify species that contributed most to the differences between two types of plots.

PERMANOVA was also employed to investigate differences in the species composition and vegetation parameters between two seasons, spring and summer. It was performed as described earlier (with site identifier as a random effect and Euclidean and Bray–Curtis distances for vegetation parameters and species abundances, respectively) separately for invaded plots and uninvaded plots.

The relationship between the occurrence of species within *R. japonica* patches and habitat conditions was determined with distance-based linear models (DistLM) (Anderson *et al*. 2008). Explanatory variables were soil physicochemical properties (16 variables, including the thickness of the organic horizon) and the size of the *R. japonica* patch. Forward selection procedure and the adjusted *R*^2^-value criterion were used to obtain the best model explaining the variability in the species composition of resident plants in the *R. japonica* plots.

To visualize the differences between the two types of plots, a principal coordinates analysis (PCoA) ordination was generated, wherein plots were symbol-coded according to plot type. The differences between the two seasons were visualized in the same way. PCoA was performed after each PERMANOVA on exactly the same data as the corresponding PERMANOVA. To visualize the relationship between habitat conditions and the species composition of resident plants in the *R. japonica* plots, distance-based redundancy analysis (dbRDA) was used.

As a rule, species with a low frequency, i.e. occurring on <10 % of plots, were excluded from multivariate analyses. An exception was made for species data from *R. japonica* plots; due to the small number of species in these plots, no species were excluded.

All multivariate analyses were carried out using PRIMER 7 with the PERMANOVA+ package (Anderson *et al*. 2008). For the purpose of interpreting the results of these analyses, univariate tests, including non-parametric two-sample paired tests, were performed using PAST 3.14 (Hammer *et al*. 2001).

## Results

### Soil properties in invaded and uninvaded plots

**Figure 1.**
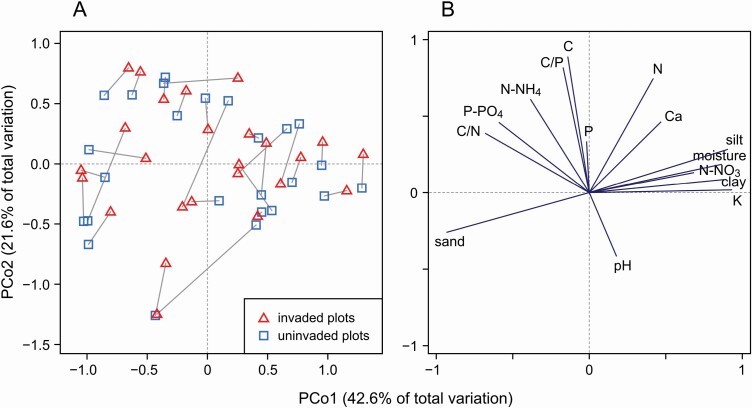
The results of principal coordinates analysis (PCoA) for soil physicochemical properties determined for 50 study plots (25 sites); the analysis based on Euclidean distances. The left diagram (A) shows the position of invaded plots (red triangles) and uninvaded plots (blue squares) in the ordination space; plots within the same site (from the same pair) were connected with a grey line. The right diagram (B) shows the projection of soil variables onto the ordination space.

According to PERMANOVA, the *R. japonica* (invaded) plots did not differ from the resident vegetation (uninvaded) plots in terms of soil physicochemical properties (pseudo-*F* = 1.1, *P* = 0.334). This lack of differences was also confirmed by univariate tests (paired *t*-tests; **see****Supporting Information—Table S1**) – no statistically significant result was obtained for any of the variables (note that the O horizon thickness was excluded from the above statistical analyses because it was not measured in uninvaded plots). Interestingly, the soil physicochemical properties varied widely in this study (Fig. 1), but the predominant source of this variability was the site location, not the type of plot. The PCoA results (Fig. 1B) showed that the main environmental gradient was soil type variability, which can be inferred from the variables related to the PCo1 axis (sand, silt, clay, moisture, K). The second important gradient reflected the content of organic matter, as evidenced by the high correlation of C and N with the PCo2 axis. Figure 1A indicates that the plots in pairs differed mainly in position along the PCo2 axis. However, these differences were small and inconsistent (many invaded plots had higher PCo2 scores than the corresponding uninvaded plots, but, in several cases, the opposite was true).

### Resident plant communities in invaded and uninvaded plots

A total of 83 species of resident (other than *R. japonica*) vascular plants were found in the studied plots (*N* = 50), of which one—*Allium ursinum—*is protected by Polish law. There were 28 species shared by both types of plots, 51 species unique to the uninvaded plots and only 4 species unique to the invaded plots. Among the latter, *Echinocystis lobata* was the most common; it occurred in four plots with abundance from 1 to 7. The number of species (species richness) ranged from 0 to 9, averaging 4, in the invaded plots, while it ranged from 4 to 24, averaging 11, in the uninvaded plots. Based on the lists of species and the results presented below, vegetation of *R. japonica* plots was classified as *Reynoutrietum japonicae* Görs et Müller in the Görs 1975 association (the *Galio-Urticetea* Passarge ex Kopecký 1969 class).

**Figure 2.**
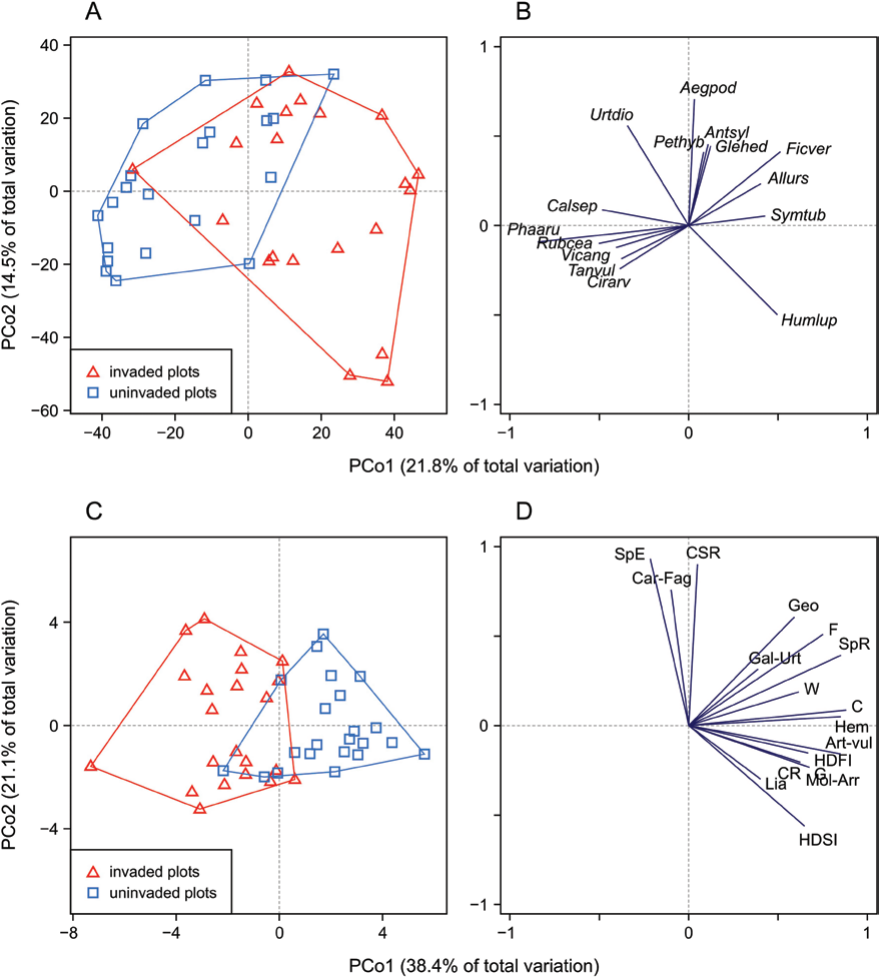
The results of principal coordinates analysis (PCoA) for data on the occurrence of resident plant species (A–B) in 42 study plots (21 sites) and vegetation parameters (C–D) calculated based on species data for 44 study plots (22 sites); the analysis based on Bray-Curtis distances (A–B) and on Euclidean distances (C–D). Some pairs of plots were excluded from the analysis due to ‘empty’ plots (see the text for explanation). The left diagrams (A and C) show the position of invaded plots (red triangles) and uninvaded plots (blue squares) in the ordination space; plots of the same type were enveloped. The right diagrams (B and D) show the projection of plant species (B) and vegetation parameters (D) onto the ordination space; for clarity, only variables that correlate best (*r*>0.4) with the PCoA axes were displayed. Explanation of species names abbreviations: *Aegpod—Aegopodium podagraria*, *Allurs—Allium ursinum*, *Antsyl—Anthriscus sylvestris*, *Calsep—Calystegia sepium*, *Cirarv—Cirsium arvense*, *Ficver—Ficaria verna*, *Glehed—Glechoma hederacea*, *Humlup—Humulus lupulus*, *Pethyb—Petasites hybridus*, *Phaaru—Phalaris arundinacea*, *Rubcea—Rubus caesius*, *Symtub—Symphytum tuberosum*, *Tanvul—Tanacetum vulgare*, *Urtdio—Urtica dioica*, *Vicang—Vicia angustifolia*. Explanation of vegetation parameters abbreviations: SpR—species richness, SpE—spring ephemerals, C—competitors, CR—competitive-ruderals, CSR—mixed strategists, Geo—geophytes, Hem—hemicryptophytes, Lia—lianas, F—forbs, G—graminoids, W—woody plants, Art-vul—*Artemisietea vulgaris*, Car-Fag—*Carpino-Fagetea*, Gal-Urt—*Galio-Urticetea*, Mol-Arr—*Molinio-Arrhenatheretea*, HDFI—herb-layer disturbance frequency index, HDSI—herb-layer disturbance severity index.

PERMANOVA showed a highly significant difference between the two types of plots in the plant species composition (pseudo-*F* = 9.9, *P* < 0.001). In the PCoA diagram, it is manifested by a clear shift of points representing the invaded plots to the right (i.e. along the PCo1 axis) in relation to those representing the uninvaded plots (Fig. 2A). According to PCoA (Fig. 2B), SIMPER analysis (Table 1) and univariate tests **[see****Supporting Information—Table S2****]**, two groups of species were responsible for this shift: one included plants associated with invaded plots, such as *Humulus lupulus*, *Symphytum tuberosum* and *Ficaria verna* (they were able to coexist with *R. japonica*), the other included plants associated with uninvaded plots, primarily *Phalaris arundinacea* and *Rubus caesius* (they were common and even dominant components of resident vegetation while being scarce or almost absent in the *R. japonica* patches). The results of the analyses also showed that two species, *Aegopodium podagraria* and *Urtica dioica*, were relatively constant components of the plant communities of both types of plots; they contributed to the within-plot type similarity (Table 1) and were related to high scores on PCo2 axis (Fig. 2B).

**Table 1.** Resident plant species that contribute most to the dissimilarity (Dis) between invaded plots (I) and uninvaded plots (U), and those that contribute to the similarity (Sim) among plots within a given plot type, according to the SIMPER analysis. For each species, its mean abundances (based on squre-root transformed cover-abundance values expressed on the 12-point scale; see the text) in both plot types and its percentage contributions are shown. The average dissimilarity between the two plot types is 84.9 %, while the average similarities of the invaded plots and uninvaded plots are 18.6 and 35.2 %, respectively. The mean abundances of species distinguishing a given plot type are in bold.

Species	Mean abundance		Contribution (%)		
	I	U	Dis (I/U)	Sim (I)	Sim (U)
*Phalaris arundinacea*	0.08	**2.22**	12.94		26.57
*Rubus caesius*	0.33	**1.67**	8.31		14.44
*Aegopodium podagraria*	**0.72**	**1.48**	7.51	14.43	11.31
*Urtica dioica*	**0.56**	**1.39**	6.58	21.82	12.13
*Calystegia sepium*	0.52	**1.01**	6.00		8.12
*Petasites hybridus*	0.14	1.01	4.75		
*Humulus lupulus*	**0.79**	0.17	4.51	16.79	
*Chaerophyllum aromaticum*	0.32	0.77	4.43		
*Symphytum tuberosum*	**0.75**	0.26	4.30	14.48	
*Agrostis stolonifera*	0	0.68	4.27		
*Anthriscus sylvestris*	0.27	0.72	3.95		
*Ficaria verna*	**0.59**	0.39	3.83	6.95	

When the vegetation parameters (Table 2) were subjected to PERMANOVA, the difference between the two plot types turned out to be more pronounced than for the species data (pseudo-*F* = 17.8, *P* < 0.001). This result was well visualized by the PCoA diagram (Fig. 2C); points representing the invaded and uninvaded plots overlapped very little. According to PCoA, the species richness of resident plants and most of the remaining resident plant community traits (including the numbers of colonizers, graminoids, forbs, hemicryptophytes, geophytes and representatives of the *Artemisietea vulgaris* and *Molinio-Arrhenatheretea* classes, as well as disturbance indices) were associated with the uninvaded plots (Fig. 2D). Three variables, spring ephemerals, mixed (CSR) strategists and *Carpino-Fagetea* class representatives, were characterized by a different pattern – they were completely independent of the main gradient (PCo1 axis), which means that they did not distinguish any type of plot.

**Table 2.** Resident plant community parameters (means and standard deviations) determined for the invaded and uninvaded plots. Plant traits that were rarely represented (less than 3 records) were not shown. Except for HDFI and HDSI, variables are counts of species, both total (species richness) and representing particular functional traits. Plot types were compared using Wilcoxon signed-rank test (*N* = 25). Significant *P*-values (<0.05) are in bold. HDFI – herb-layer disturbance frequency index; HDSI – herb-layer disturbance severity index.

Variable	Invaded	Uninvaded	*P*-value
Species richness	3.8 (2.6)	11.2 (5.2)	**0.000**
Spring ephemerals	0.8 (1.2)	0.4 (1.0)	**0.023**
Graminoids	0.0 (0.2)	1.8 (1.0)	**0.000**
Forbs	2.9 (1.9)	5.3 (3.2)	**0.001**
Woody plants	0.3 (0.5)	0.9 (0.9)	**0.005**
C (competitors)	2.0 (1.5)	6.0 (2.4)	**0.000**
CR (competitive-ruderals)	0.4 (0.7)	1.0 (1.0)	**0.001**
CSR (mixed strategists)	0.8 (1.0)	0.8 (1.0)	0.934
*Artemisietea vulgaris*	0.7 (0.8)	2.4 (1.7)	**0.000**
*Carpino-Fagetea*	1.0 (1.1)	0.5 (0.7)	**0.047**
*Galio-Urticetea*	1.2 (1.0)	1.8 (1.5)	0.064
*Molinio-Arrhenatheretea*	0.1 (0.3)	1.6 (1.4)	**0.000**
Geophytes	1.4 (1.3)	3.2 (1.2)	**0.000**
Hemicryptophytes	1.2 (1.0)	3.5 (1.7)	**0.000**
Lianas	0.7 (0.7)	0.8 (0.8)	0.592
Therophytes	0.4 (0.6)	0.5 (0.7)	0.305
HDFI	−0.62 (0.11)	−0.50 (0.7)	**0.000**
HDSI	0.34 (0.10)	0.42 (0.05)	**0.002**

### Between-season variability of invaded and uninvaded plant communities

According to PERMANOVA, the difference between spring and summer in the species composition of resident plants in invaded plots was at the verge of statistical significance (pseudo-*F* = 1.8, *P* = 0.067). For the uninvaded plots, this difference was statistically significant but weak (pseudo-*F* = 3.7, *P* = 0.034).

**Figure 3.**
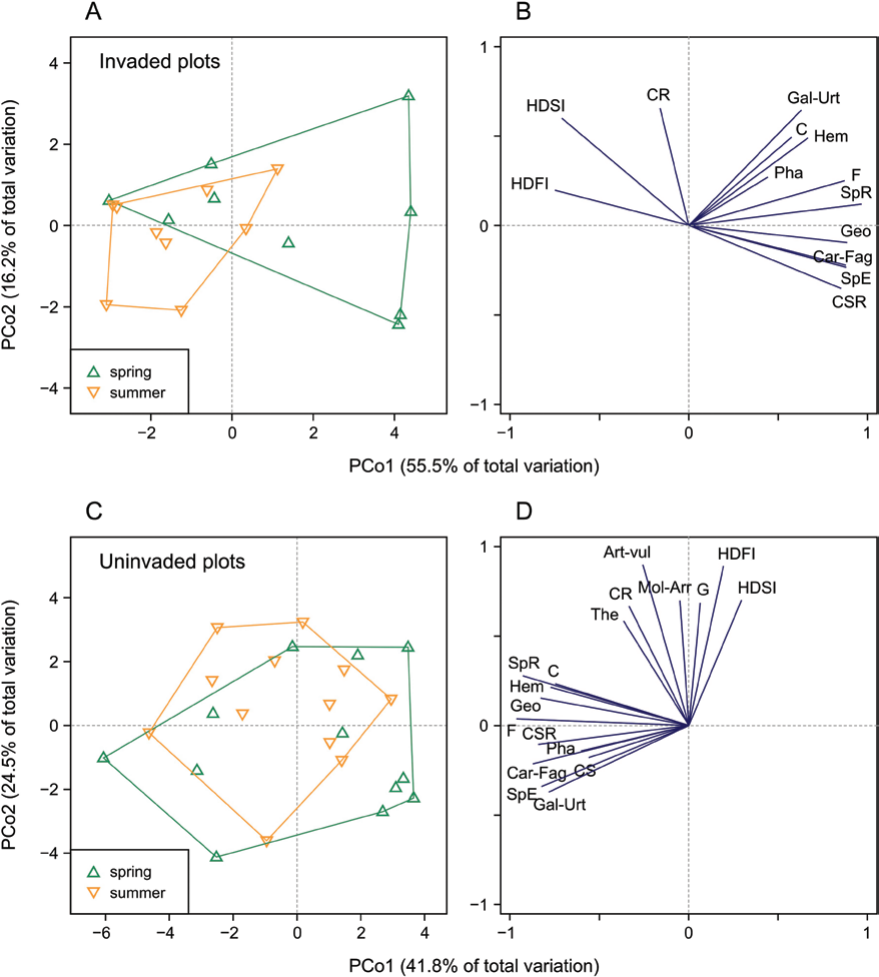
The results of principal coordinates analysis (PCoA) for vegetation parameters calculated based on species data collected in the spring (up-pointing green triangle) and summer (down-pointing orange triangle) seasons from 9 invaded plots (A–B) and 12 uninvaded plots (C–D). The analysis based on Euclidean distances. Some pairs of plots were excluded from the analysis due to ‘empty’ plots (see the text for explanation). The left diagrams (A and C) show the position of plots, separately for spring and summer seasons, in the ordination space; plots of the same type were enveloped. The right diagrams (B and D) show the projection of vegetation parameters onto the ordination space; for clarity, only variables that correlate best (*r* > 0.4) with the PCoA axes were displayed. Explanation of abbreviations: SpR—species richness, SpE—spring ephemerals, C—competitors, CR—competitive-ruderals, CS—stress-tolerant competitors, CSR—mixed strategists, Geo—geophytes, Hem—hemicryptophytes, Pha—phanerophytes, The—therophytes, F—forbs, G—graminoids, Art-vul—*Artemisietea vulgaris*, Car-Fag—*Carpino-Fagetea*, Gal-Urt—*Galio-Urticetea*, Mol-Arr—*Molinio-Arrhenatheretea*, HDFI—herb-layer disturbance frequency index, HDSI—herb-layer disturbance severity index.

In contrast, PERMANOVA performed on vegetation parameters (Table 3) showed that the two seasons were clearly different from each other, but the pattern depended on the type of plot. In the case of invaded plots, the difference (pseudo-*F* = 5.1, *P* = 0.017; Fig. 3A) was mainly due to the fact that most species occurred exclusively or more abundantly in spring than in summer (Fig. 3B). This is especially true of spring ephemerals, such as *Ficaria verna*, *Anemone nemorosa* and *Allium ursinum*, which are geophytes belonging to the *Carpino-Fagetea* class. As shown by the PCoA diagram (Fig. 3B), disturbance indices (HDFI and HDSI) were the only parameters with generally higher scores in the summer surveys than in the spring surveys. In the case of uninvaded plots, the difference (pseudo-*F* = 4.5, *P* = 0.008) was less pronounced and other variables contributed to it. The points representing the summer surveys (occupying slightly higher positions in the PCoA diagram; Fig. 3C) were associated with the presence of a greater number of species, with these species being competitive-ruderals, graminoids, therophytes and representatives of the *Artemisietea vulgaris* and *Molinio-Arrhenatheretea* classes (Fig. 3D). Higher values of disturbance indices were also associated with the summer surveys. Spring ephemerals, which were important in the case of the invaded plots, did not play a role in differentiating the two seasons.

**Table 3.** Resident vegetation parameters (means and standard deviations) determined for spring and summer separately for the invaded and uninvaded plots. Plant traits that were rarely represented (less than 3 records) were not shown. Except for herb layer cover, HDFI and HDSI, variables are counts of species, both total (species richness) and representing particular functional traits. *Values estimated for total vegetation (including *R. japonica*). HDFI – herb-layer disturbance frequency index, HDSI – herb-layer disturbance severity index. Seasons were compared using Wilcoxon signed-rank test (*N* = 12). Significant *P*-values (<0.05) are in bold.

Variable	Invaded			Uninvaded		
	Season		*P*-value	Season		P-value
	Spring	Summer		Spring	Summer	
Herb layer cover (%)	31 (27)*	100 (0)*	**0.000**	90 (11)	99 (4)	**0.020**
Species richness	3.3 (2.9)	1.1 (1.2)	**0.015**	5.4 (3.5)	6.7 (2.8)	0.125
Spring ephemerals	1.4 (1.9)	0.0 (0.0)	**0.027**	1.4 (2.2)	0.5 (0.8)	0.066
Graminoids	0.2 (0.6)	0.0 (0.0)	0.317	1.6 (0.7)	2.3 (1.1)	0.054
Forbs	2.9 (2.5)	0.8 (0.8)	**0.017**	3.8 (3.3)	4.8 (2.3)	0.131
C (competitors)	1.6 (1.6)	0.8 (1.0)	**0.023**	4.3 (1.4)	6.1 (2.1)	**0.016**
CR (competitive-ruderals)	0.3 (0.7)	0.3 (0.5)	0.705	0.3 (0.5)	0.5 (0.7)	0.414
CS (stress-tolerant competitors)	0.3 (0.6)	0.0 (0.0)	0.180	0.3 (0.6)	0.8 (0.9)	0.102
CSR (mixed strategists)	1.2 (1.7)	0.0 (0.0)	**0.026**	1.3 (2.0)	0.5 (0.9)	0.070
*Artemisietea vulgaris*	0.1 (0.3)	0.1 (0.3)	1	1.0 (1.1)	1.8 (1.0)	**0.029**
*Carpino-Fagetea*	1.4 (1.7)	0.2 (0.4)	**0.016**	1.5 (2.5)	0.6 (1.0)	0.056
*Galio-Urticenea*	1.5 (1.4)	0.8 (0.9)	**0.023**	2.3 (2.1)	3.1 (2.2)	**0.008**
*Molinio-Arrhenatheretea*	0.1 (0.3)	0.0 (0.0)	0.320	1.2 (0.7)	2.5 (1.6)	**0.013**
Geophytes	1.7 (2.0)	0.5 (0.7)	0.065	2.5 (1.4)	2.8 (1.4)	0.623
Hemicryptophytes	0.1 (0.3)	0.3 (0.5)	**0.016**	2.8 (1.9)	4.3 (1.8)	**0.018**
Phanerophytes	0.2 (0.4)	0.2 (0.4)	1	0.8 (0.9)	0.7 (0.8)	0.563
Therophytes	0.1 (0.3)	0.0 (0.0)	0.320	0.7 (0.8)	0.5 (0.5)	0.317
HDFI	−0.57 (0.05)	−0.55 (0.06)	0.310	−0.54 (0.10)	−0.51 (0.07)	0.136
HDSI	0.37 (0.09)	0.39 (0.09)	0.398	0.38 (0.09)	0.41 (0.05)	0.084

### Relationship between plant species composition and habitat properties in invaded plots

**Figure 4.**
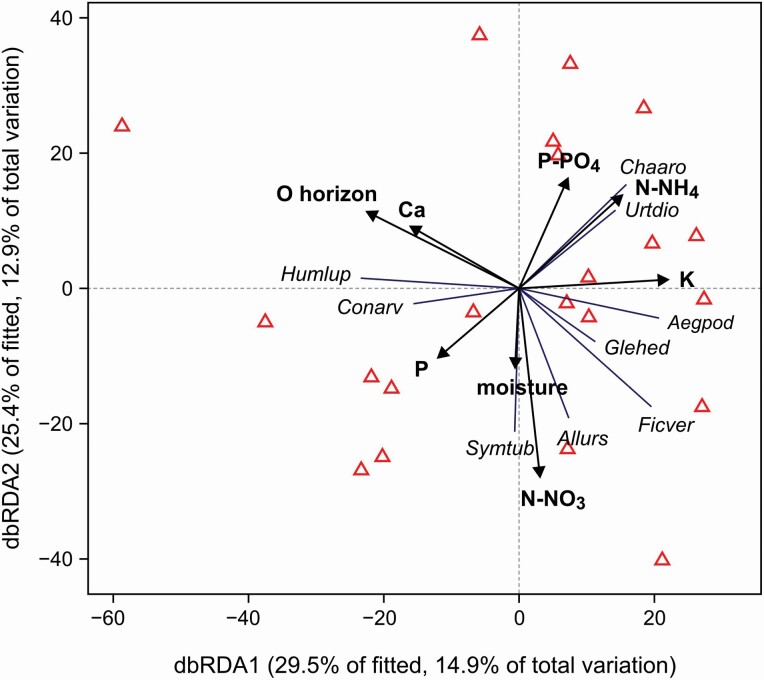
The results of distance-based redundancy analysis (dbRDA) showing the relationship between habitat properties (forward-selected according to the adjusted *R*^2^-value criterion) and resident plant species occurrence for the invaded plots (red triangles). The analysis based on Bray-Curtis distances.

DistLM analysis selected eight habitat variables to explain the species composition of resident plants in invaded plots; they were shown in the dbRDA diagram (Fig. 4). Among these variables, the most important were: the O horizon thickness (averaging 5.6 ± 3.4 cm), related to the dbRDA1 axis, and N-NO_3_, related to the dbRDA2 axis. Most of the species were on the right side of the diagram—they were negatively related to the O horizon thickness (Fig. 4). Only lianas, *Humulus lupulus* and *Convolvulus arvensis*, showed the opposite pattern. Species avoiding a thick organic layer formed two groups: the bottom of the diagram was occupied by spring ephemerals (*Symphytum tuberosum*, *Allium ursinum*, *Ficaria verna*), and the top of the diagram by nitrophilous species with high competitive abilities, i.e. *Urtica dioica*, *Chaerophyllum aromaticum* and, partially, *Aegopodium podagraria*. Variables related to the dbRDA2 axis suggest that the availability of potentially limiting nutrients, N and P, might determine the occurrence of these species in invaded plots.

## Discussion

Our study is the first to link the diversity and species composition of resident vegetation affected by invasive *R. japonica* with soil physicochemical properties, which, combined with extensive sampling taking into account seasonal variability, allowed for strong inferences. As expected, the invasion of *R. japonica* caused profound changes in resident plant communities. It not only dramatically reduced their species diversity, leading to a significant homogenization of the plant cover, but also strongly influenced community structure (not all resident plants responded equally to the pressure of the invader).

The basic mechanism responsible for the considerable reduction of species diversity at invaded sites is the fast and extensive clonal growth of *R. japonica* and its strong ability to spread and form near-monoculture stands, which results in limitations of light availability to other plants (Tokarska-Guzik *et al*. 2006; Aguilera *et al*. 2010; Maurel *et al*. 2010; Moravcová *et al*. 2011). However, the intensity of *R. japonica* impact depends on the soil and vegetation types, season and the presence of several resident species with considerable resistance to invasion.

Our previous study showed that *R. japonica* biomass was qualitatively different from that of resident vegetation; it was characterized by significantly lower N, P and K contents, higher C and Ca contents, and higher C/N and C/P ratios (Stefanowicz *et al*. 2020). However, this did not result in differences in the nutrient stoichiometry of the organo-mineral horizon (horizon A) between the invaded and uninvaded plots as illustrated by PERMANOVA and PCoA results. This means that the indirect influence of the invader by modifying the soil environment can be considered negligible. The only soil parameter that turned out to be significant for the resident plants was the thickness of the organic (O) horizon. The accumulation of organic matter in the topsoil, which is the result of the large amount of biomass produced by *R. japonica* and the low rate of its decomposition, limited the occurrence of most species observed in the *R. japonica* plots, including spring ephemerals and highly competitive species, e.g. *Aegopodium podagraria* and *Urtica dioica*. Only lianas, especially *Humulus lupulus*, seemed to tolerate the organic matter accumulation. Since high values of disturbance indices (HDFI and HDSI) were associated with a thick O horizon, the hardly decomposing organic matter produced by *R. japonica* should be considered as a kind of environmental stress, which, together with the reduced availability of light, has a strong impact on resident vegetation. Our results are in line with the findings of other studies where large amounts of *R. japonica* organic matter were harmful to the establishment of most vascular plants and the performance of microbial populations (Aguilera *et al*. 2010; Maurel *et al*. 2010; Mincheva *et al*. 2014).

It is well known that litter affects plant community structure and dynamics (Facelli and Pickett 1991). In response to this factor, specific plant strategies have evolved, including adaptive traits to cope with the accumulation of dense litter mats (Grime 2001). For example, important components of plant communities in temperate deciduous forests are geophytes and hemicryptophytes, whose shoots, thanks to their specific morphology, can penetrate thick layers of litter occurring in early spring. It is possible that the presence of spring ephemerals, such as *Allium ursinum*, *Anemone nemorosa*, *Ficaria verna* and *Symphytum tuberosum* (which are geophytes), in the *R. japonica* plots is possible not only because of their ability to use a short period of high light availability but also because of their adaptation to growth under thick litter mat conditions (although they seem to avoid places with the thickest O horizon as shown earlier).

Many field and laboratory studies indicated that *R. japonica* might affect resident plants through indirect allelopathy, the negative effects of which are mediated by the soil microbiome (Murrell *et al*. 2011; Parepa *et al*. 2013b; Tanner and Gange 2013). In contrast, resident species typically have the opposite effect. *Calamagrostis epigejos*, *Phalaris arundinacea* and *Urtica dioica* were among the most important components of the resident plant communities in our study. These three species are known to be beneficial for soil microorganisms (Valé *et al*. 2005; Stefanowicz *et al*. 2015, 2016; Espenberg *et al*. 2016; Woch *et al*. 2018). For example, *Phalaris arundinacea*, which dominated most of the uninvaded plots, produces large amounts of biomass with properties promoting soil microbial populations (Espenberg *et al*. 2016). According to our recent study (Stefanowicz *et al*. 2021), soils from invaded plots had a much lower microbial biomass than soils from uninvaded plots. It is, therefore, possible that *R. japonica* affects resident vegetation not only through direct competition but also indirectly, through a negative effect on microbial communities (reducing microbial performance through both allelopathy and displacement of species favoring soil microorganisms).

According to some authors (Tharayil *et al*. 2013; Dommanget *et al*. 2014; Mincheva *et al*. 2014; Suseela *et al*. 2016), phenolics constitute the allelopathic weapon of *R. japonica*. Indeed, our recent study showed that the content of phenolics in the *R. japonica* tissues, especially in the rhizomes and leaves, was very high (Stefanowicz *et al*. 2021). However, it did not result in high concentrations of these compounds in the soil; these concentrations were comparable to those in the uninvaded plots. It is possible that the persistence of *R. japonica* allelochemicals is low, and their impact is seasonal or restricted to the organic layer and mineral soil surface. The slow release of phenolic compounds from the leaf litter (Lavoie 2017) suppresses the germination of the seeds of other species (Moravcová *et al*. 2011; Šerá 2012; Vrchotová and Šerá 2018), which generally takes place in the upper layers of the soil profile. This mechanism may be selective; *R.* × *bohemica*, which is a close *R. japonica* relative, exhibits allopathic effects on native forbs but not on grasses (Murrell *et al*. 2011).

Altering resource availability, e.g. by releasing both nutrients and secondary metabolites into the soil, is another strategy to facilitate invasion (Davis *et al*. 2000; Dawson *et al*. 2012; Tharayil *et al*. 2013; Abgrall *et al*. 2018). Tharayil *et al*. (2013) revealed that *R. japonica* phenolic compounds slow down soil N cycling and reduce the accumulation of inorganic N at the start of the growing season, causing a deficiency of available nitrogen for the resident species during this period. This phenomenon may additionally explain the high survival rate of spring ephemerals that are geophytes. Geophytes have an adaptive strategy to store carbohydrates in underground organs (rhizomes, tubers) for fast growth in spring when they do not yet face competition for nutrients and light (Chapin *et al*. 1990). This group of plants showed high frequency and coverage in the *R. japonica* plots. Vernal geophytes such as *Allium ursinum*, *Ficaria verna* and *Symphytum tuberosum* (see Supporting Information—Fig. S1D) can go through their full growing cycle in *R. japonica* patches, between snowmelt and the development of the invader canopy. According to the dbRDA diagram (Fig. 4), they were in the N and P availability gradient on the opposite side to the nitrophilous species (e.g. *Urtica dioica*). This suggests that they survive in the *R. japonica* patches, occupying niches associated with periodic nutrient deficiencies possibly created by the invader.

Lianas (e.g. *Humulus lupulus* and *Calystegia sepium*) are another group of species that do well under the *R. japonica* invasion. Their strategy, however, is different from that of spring ephemerals. They start to grow in spring, use withered **[see****Supporting Information—Fig. S1E****]** and then live shoots of *R. japonica* to climb, then overgrow the supporting plant **[see****Supporting Information—Fig. S1C****]**, and finally, in late summer, produce seeds, thus closing the growing cycle. Interestingly, one of the recorded liana species was *Echinocystis lobata—*an invasive plant. Apparently, it used the invasion of *R. japonica* to spread itself. This species was observed in four *R. japonica* plots, where it achieved relatively high coverages (up to 7). It should be emphasized, that its frequency and abundance is underestimated, because, when establishing the study sites, we tried to select places unaffected by other invasive plants than *R. japonica*.

To understand why certain resident species withstand the invader pressure more easily than others, it is helpful to refer to the coexistence theory. According to this theory, spatial or temporal separation of niches releases species from competition (Anten and Hirose 1999; Engelhardt and Anderson 2011; Godoy and Levine 2014; Wolkovich and Cleland 2014). Spring ephemerals and lianas can coexist with *R. japonica* because they have different strategies of resource use: the former choose an earlier part of the growing season to go through the full growing cycle (temporal separation), the latter use their climbing abilities to lift the foliage above the invader canopy (spatial separation). In the case of resident species occupying a similar niche as the invasive species, those with a high potential to compete for resources have a chance of survival (Mayfield and Levine 2010; Gallien *et al*. 2015; Fried *et al*. 2019). *Urtica dioica* and *Aegopodium podagraria* seem to be such species. Although *R. japonica* significantly reduced their abundances, it was not able to completely replace them. According to (Chytrý 2007), these species are quite a constant element of the *Reynoutrietum japonicae* association.

Another factor that may determine the species composition of resident plants in the *R. japonica* plots is the type of habitat, i.e. broadly understood environmental conditions. This is indicated by the lack of spring ephemerals within fallows, i.e. in drier and more sandy sites, far from the floodplains where these species occur naturally.

The way *R. japonica* displaces other species and dominates the community is not unique in the plant world. For example, *Microstegium vimineum* (native to east Asia, invasive in North America), *Phragmites australis* (native to Europe, invasive in North America) and *Pteridium aquilinum* (native to Poland, invasive in Australia, Great Britain, New Zealand and North America) can also dramatically reduce the diversity and change the structure of resident vegetation (Chambers *et al*. 1999; Gordon *et al*. 1999; Brewer 2011). These fast-growing species, with persistent and copious rhizomes, tend to form dense, mono-species stands (Whitehead and Digby 1997; Brewer 2011; DeLuca *et al*. 2013). The rapid accumulation of their litter alters the physical properties of the soil and soil microbial processes (Whitehead and Digby 1997; Rooth *et al*. 2003; Strickland *et al*. 2011; Ssali *et al*. 2018). This leads to a radical change of the habitat, which directly and indirectly transforms the biocenosis (decrease in the number of species, replacement of specialists by generalists, structural homogenization). Due to the similarity of *R. japonica* to other invasive plants in terms of plant traits and the way they affect resident vegetation, the results presented in this paper may be of universal importance; for example, they might help predict the effects of other plant species invasions.

When interpreting the results of field studies, their limitations should be taken into account. These studies assume that the pre-invasive state of invaded and non-invaded plots is the same. This is not necessarily true. The invasion at a given location could be induced or facilitated by, for example, local soil disturbances or a slightly different structure of the community (e.g. lower vegetation density, different composition of species), which could also affect, apart from the invader itself, resident vegetation (MacDougall and Turkington 2005). We cannot say whether this was the case in our study because we do not know the history of the studied sites. However, it seems that the probability of pre-existing differences between plots in a pair is small. The *R. japonica* patches we investigated were quite large (ca 200 m^2^ on average). If their formation was initiated by any factor, then this factor acted rather locally, in the middle of the present patches, from where the invasive plant then spread by its own forces. Since we established the plots in pairs close to each other (to minimize environmental differences between them), the *R. japonica* plots were closer to the edge than to the middles of patches, i.e. outside the hypothetical place of disturbance. The absence of any differences in soil physicochemical properties supports this scenario.

## Conclusions

Our study showed that *R. japonica* has a strong negative effect on resident vegetation by either completely displacing or drastically reducing the abundance of many plant species. The great success in outcompeting other plants results from the multifaceted influence of the invader. This includes quickly occupying new space, limiting access to light and producing a thick layer of hard-to-decompose litter (O horizon). It seems that direct and indirect (e.g. via soil microorganisms) allelopathic effects and the control of nutrient availability may also play a role. Not all species are displaced by *R. japonica*. There are some that perform well in invaded patches and even take advantage of invasion. It seems to be determined by a combination of plant traits: Grimes’s strategy, Raunkiaer’s life form and belonging to one of the functional groups. Species that are able to coexist with *R. japonica* can be classified into three groups:

• geophytic forbs with a mixed life strategy that take advantage of the periodic (spring) release from competition (mainly for light) from *R. japonica*.
• lianas with a competitive strategy—plants that rise above *R. japonica*, using it as a support, and thus avoiding competition for light; they seem to tolerate the thick O horizon formed by the invader litter.
• hemicryptophytic forbs with a competitive strategy—plants that, like *R. japonica*, have an outstanding ability to dominate the community and create almost mono-species stands; thanks to this trait, they are able to utilize resources and, although they do not win against the invader (in terms of abundance), they cannot be completely eliminated.

## Supporting Information

The following additional information is available in the online version of this article—

**Figure S1.** (A) An example of a study site with a *Reynoutria japonica* patch (right) and resident vegetation (left). (B) An invaded plot in summer, with a fully developed, compact *R. japonica* canopy, and a floor almost devoid of other species. (C) Dense *R. japonica* canopy overgrown by a liana—Humulus *lupulus*. (D) An invaded plot in early spring, with withered and new, fast-growing annual shoots of *R. japonica* and a flowering geophyte—Symphytum *officinale*. (E) *H. lupulus* in early spring climbing up the old shoots of *R. japonica*. Phot. M. Woch.

**Table S1.** Soil physicochemical properties (means and standard deviations) for 25 invaded (*R. japonica*) and 25 uninvaded plots. Soil samples were taken from horizon A to a depth of 20 cm. According to paired *t*-tests, none of the variables differed statistically significantly (*P* > 0.05) between the two plot types.

**Table S2.** Frequency (F, the number of species records) and abundance (A, mean and standard deviation calculated from cover-abundance values expressed on the 12-point scale) of the most frequent (present in at least 10 % plots) resident plant species in 25 invaded (*R. japonica*) and 25 uninvaded plots, and selected functional traits (GLS—Grime’s life strategy, FUN—functional group, RLF—Raunkiaer’s life form, COM—belonging to one of the plant community classes).

plab035_suppl_Supplementary_Materials

## Data Availability

https://doi.org/10.18150/IMO5TH, RepOD, V1.

## Notes

**Populations & Communities. Chief Editor:** Jean Burns
