# Supplementary material for: Functional traits predict resident plant response to Reynoutria japonica invasion in riparian and fallow communities in southern Poland
Source: AoB Plants. 2021 Jun 3;13(4):plab035. doi: 10.1093/aobpla/plab035 (PMC8266581; doi:10.1093/aobpla/plab035)
Supplement: plab035_suppl_Supplementary_Materials [file plab035_suppl_supplementary_materials.pdf]

Supporting Information (SI)

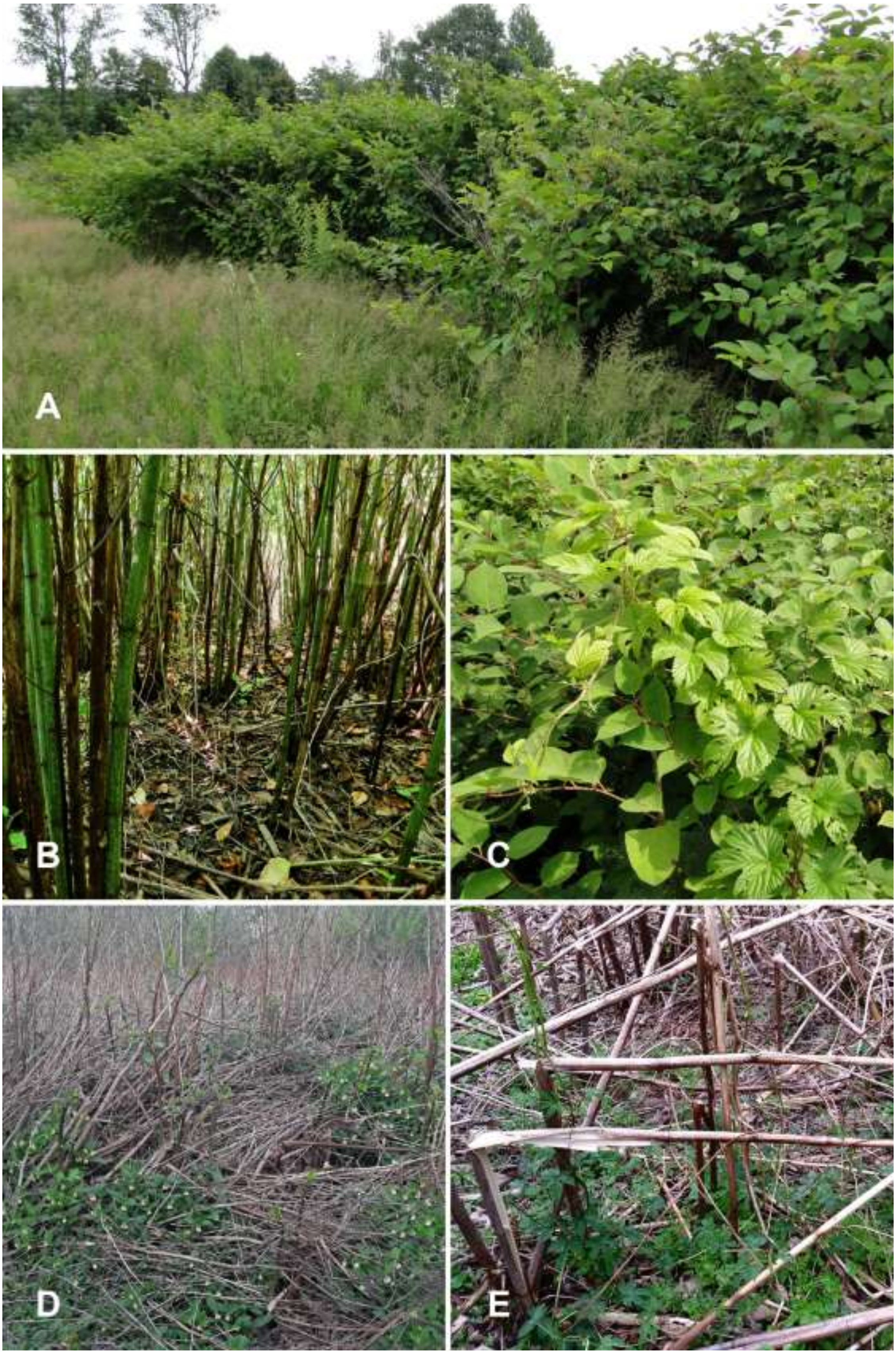

Fig. S1. A) An example of a study site with a *Reynoutria japonica* patch (right) and resident vegetation (left). B) An invaded plot in summer, with a fully developed, compact *R. japonica* canopy, and a floor almost devoid of other species. C) Dense *R. japonica* canopy overgrown by a liana – *Humulus lupulus*. D) An invaded plot in early spring, with withered and new, fast-growing annual shoots of *R. japonica* and a flowering geophyte – *Symphytum officinale*. E) *H. lupulus* in early spring climbing up the old shoots of *R. japonica*. Phot. M. Woch.

Supporting Information (SI)

Table S1. Soil physicochemical properties (means and standard deviations) for 25 invaded (*R. japonica*) and 25 uninvaded plots. Soil samples were taken from horizon A to a depth of 20 cm. According to paired *t*-tests, none of the variables differed statistically significantly (P>0.05) between the two plot types.

Variable	Invaded	Uninvaded
Sand (%)	55.3 (18.9)	54.6 (20.6)
Silt (%)	38.5 (16.2)	39.6 (17.7)
Clay (%)	6.2 (3.4)	5.9 (3.5)
Moisture (%)	16.7 (5.6)	16.9 (5.7)
pH	7.08 (0.77)	7.02 (0.69)
Organic C (%)	2.36 (1.22)	2.46 (1.26)
Total N (%)	0.14 (0.05)	0.15 (0.05)
Total P (mg kg^{-1})	174 (29)	177 (37)
Total K (g kg^{-1})	3.24 (1.54)	3.25 (1.64)
Total Ca (g kg^{-1})	3.30 (1.56)	2.76 (1.29)
N-NH_4 (mg kg^{-1})	1.75 (3.18)	1.63 (3.41)
N-NO_3 (mg kg^{-1})	9.65 (6.05)	8.62 (5.05)
P-PO_4 (mg kg^{-1})	1.86 (1.10)	2.10 (1.31)
C/N	16.2 (4.8)	16.3 (5.5)
C/P	134 (61)	138 (59)

Supporting Information (SI)

Table S2. Frequency (F, the number of species records) and abundance (A, mean and standard deviation calculated from cover-abundance values expressed on the 12-point scale) of the most frequent (present in at least 10% plots) resident plant species in 25 invaded (*R. japonica*) and 25 uninvaded plots, and selected functional traits (GLS – Grime's life strategy, FUN – functional group, RLF – Raunkiaer's life form, COM – belonging to one of the plant community classes).

Species	Invaded		Uninvaded		*P*-value	GLS	FUN	RLF	COM
	F	A	F	A					
Aegopodium podagraria	8	1.3 (2.5)	13	3.2 (3.8)	**0.014**	C	Forb	G/H	*Gal-Urt*
Agrostis stolonifera	0	0.0 (0.0)	6	0.8 (2.0)	**0.028**	CSR	Graminoid	H.	*Mol-Arr*
Allium ursinum	4	0.6 (2.0)	2	0.4 (1.8)	0.465	CSR	Forb	G	*Car-Fag*
Anemone nemorosa	2	0.4 (1.4)	1	0.2 (1.2)	0.592	CSR	Forb	G	*Car-Fag*
Anthriscus sylvestris	5	0.3 (0.7)	7	1.4 (2.9)	**0.028**	C	Forb	H	*Car-Fag*
Artemisia vulgaris	0	0.0 (0.0)	6	0.5 (1.2)	**0.026**	C	Forb	H	*Art vul*
Calamagrostis epigejos	0	0.0 (0.0)	8	3.0 (4.6)	**0.011**	C	Graminoid	G/H	*Epi-Ang*
Calystegia sepium	5	1.0 (2.4)	12	1.6 (2.0)	0.220	C	Forb	G/H, L	*Gal-Urt*
Chaerophyllum aromaticum	5	0.4 (1.0)	11	1.1 (1.8)	**0.040**	C	Forb	H	*Gal-Urt*
Cirsium arvense	0	0.0 (0.0)	6	1.0 (2.0)	**0.027**	C	Forb	G	*Art vul*
Convolvulus arvensis	1	0.1 (0.2)	5	0.4 (0.9)	0.066	CR	Forb	G/H, L	*Art vul*
Echinocystis lobata	4	0.5 (1.5)	0	0.0 (0.0)	0.068	CR	Forb	T, L	*Gal-Urt*
Ficaria verna	6	1.1 (2.4)	4	0.8 (2.0)	**0.040**	CSR	Forb	G	*Car-Fag*
Galium aparine	3	0.2 (0.4)	6	0.3 (0.5)	0.366	CR	Forb	T/H	*Gal-Urt*
Heracleum sphondylium	1	0.1 (0.4)	5	0.6 (1.4)	0.071	C	Forb	H	*Mol-Arr*
Humulus lupulus	7	1.7 (3.0)	2	0.3 (1.4)	**0.018**	C	Forb	H, L	*Aln glu*
Petasites hybridus	2	0.2 (0.8)	7	2.6 (4.4)	**0.018**	CS	Forb	G/H	*Gal-Urt*
Phalaris arundinacea	1	0.1 (0.6)	17	6.8 (5.3)	**0.000**	C	Graminoid	G/H	*Mol-Arr*
Rubus caesius	6	0.3 (0.6)	14	3.8 (4.2)	**0.001**	C	Woody	N	*Art vul*
Rubus idaeus	0	0.0 (0.0)	6	0.5 (1.2)	**0.026**	C	Woody	N	*Epi-Ang*
Symphytum tuberosum	8	1.5 (2.7)	4	0.4 (1.2)	**0.011**	CSR	Forb	G	*Car-Fag*
Urtica dioica	10	0.6 (1.0)	15	2.8 (2.9)	**0.005**	C	Forb	H	*Art vul*

Explanation of abbreviations: C – competitor, S – stress-tolerator, R – ruderal, CR – competitive-ruderal, CSR – mixed strategist, G – geophyte, H – hemicryptophyte, L – liana, N – nanophanerophyte, T – therophyte, *Aln glu – Alnetea glutinosae*, *Art vul – Artemisietea vulgaris*, *Car-Fag – Carpino-Fagetea*, *Epi-Ang – Epilobietea angustifolii*, *Gal-Urt – Galio-Urticetea*, *Mol-Arr – Molinio-Arrhenatheretea*. Significant *P*-values ($p<0.05$) from Wilcoxon signed-rank tests are in bold.
